# Cerebral Phaeohyphomycosis in a Patient with Neurosarcoidosis on Chronic Steroid Therapy Secondary to Recreational Marijuana Usage

**DOI:** 10.1155/2013/191375

**Published:** 2013-02-21

**Authors:** Preetam Gongidi, Debkumar Sarkar, Eric Behling, Joshua Brody

**Affiliations:** ^1^Department of Radiology, Cooper University Hospital, Cooper Medical School of Rowan University, One Cooper Plaza, B23, Camden, NJ 08103, USA; ^2^Department of Pathology, Cooper University Hospital, Cooper Medical School of Rowan University, One Cooper Plaza, B23, Camden, NJ 08103, USA

## Abstract

Cerebral phaeohyphomycosis is often a fatal disease that typically takes a hematogenous spread after inhalation or accidental skin inoculation of pathogens. We present a patient with a history of heavy marijuana smoking while being on chronic steroid therapy for treatment of neurosarcoidosis who was found to have multiple brain abscesses from *Curvularia* sp. This is a ubiquitous soil-dwelling dematiaceous fungus that is generally thought to affect solely plants, but there is increasing evidence in the literature of it affecting humans and animals. We review the radiographic findings of neurosarcoidosis and cerebral phaeohyphomycosis as well as the pathophysiology of dematiaceous fungi infections.

## 1. Introduction

Neurosarcoidosis has a wide spectrum of clinical presentations including cranial nerve deficits and seizures. The imaging findings include enhancing parenchymal and leptomeningeal lesions often involving the skull base. Patients with neurosarcoidosis are often treated with steroids which can leave them immunocompromised and susceptible to infections. We present a patient with a history of heavy marijuana smoking while being on chronic steroid therapy for treatment of neurosarcoidosis who was found to have multiple brain abscesses from *Curvularia* sp. This is a dematiaceous fungus that very rarely results in cerebral abscesses. We review the radiographic findings of neurosarcoidosis and cerebral phaeohyphomycosis as well as the pathophysiology of dematiaceous fungi infections.

## 2. Case Report

A 37-year-old male presented with a complaint of a right occipital headache following a witnessed generalized tonic-clonic seizure. He complained of right-sided hearing loss. He denied having fevers, blurred vision, photophobia, loss of consciousness, or vomiting. The patient's medical history included recently diagnosed sarcoidosis and chronic steroid use. His social history was significant for chronic heavy marijuana use, alcohol dependence, but no intravenous drug use. 

**Figure 1:**
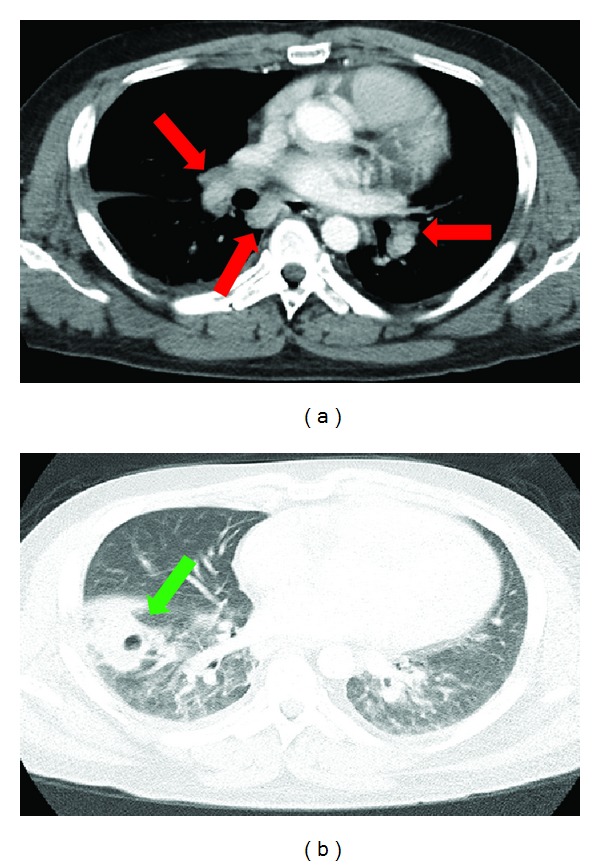
Axial contrast-enhanced CT of the chest demonstrates bilateral soft tissue densities (red arrows) consistent with bilateral hilar adenopathy (a). Opacity in right lower lobe with air-fluid level (green arrow) is consistent with pulmonary abscess (b).

**Figure 2:**
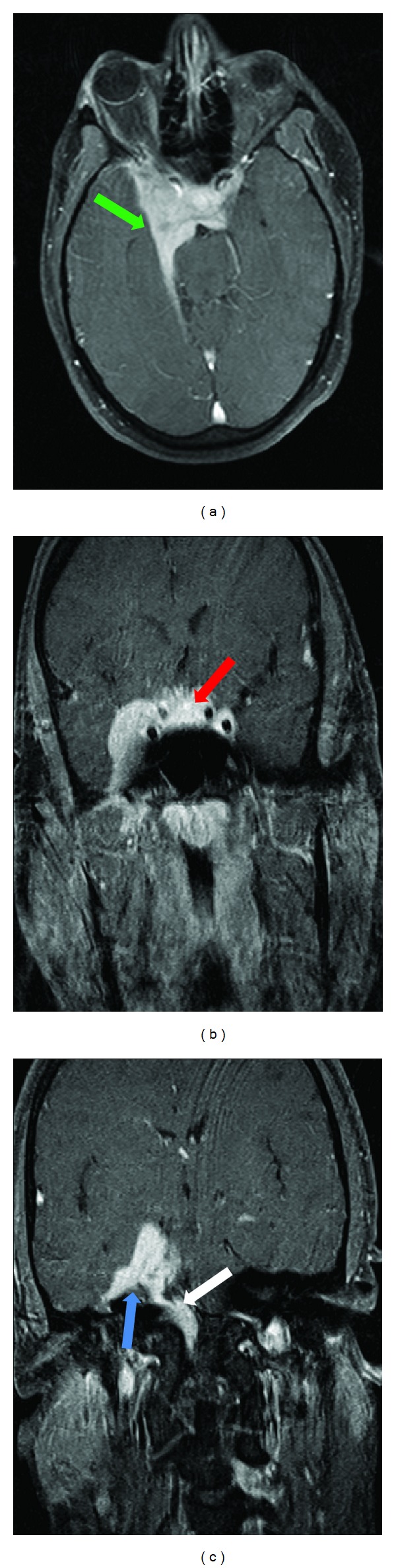
Axial (a) and coronal (b, c) T1-weighted fat suppressed images following administration of 15 mL intravenous gadolinium. There is diffuse enhancement within the skull base (green arrow), cavernous sinus (red arrow), internal auditory canal (blue arrow), prepontine cistern, and inferior extension of the enhancing lesion (white arrow) into the brainstem characteristic of granulomatous changes in neurosarcoidosis.

A chest CT demonstrated soft tissue densities in the hilar regions bilaterally consistent with bilateral hilar adenopathy characteristic of sarcoidosis. In addition there is an opacity with an air-fluid level in the right lower lobe consistent with a pulmonary abscess (Figure 1). An MRI of the brain revealed abnormal soft tissue structures in the skull base. The contrast-enhanced T1-weighted fat suppressed images showed large diffuse areas of enhancement within the skull base, with enhancement of parenchyma, leptomeninges, and dura predominantly in the cavernous sinus, internal auditory canal, prepontine cistern, optic chiasm, and optic tract regions (Figure 2). There was also involvement of the pituitary and hypophyses. The diffuse enhancing parenchymal and leptomeningeal lesions were most consistent with neurosarcoidosis. The patient was subsequently placed on long-term steroid therapy. 

**Figure 3:**
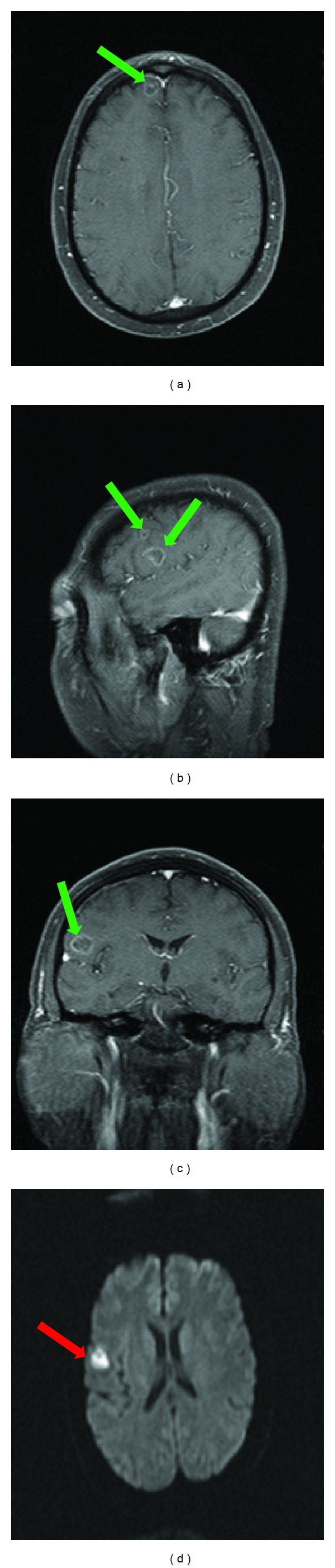
Axial (a), sagittal (b), and coronal (c) T1-weighted fat suppressed images following administration of 15 mL of intravenous gadolinium demonstrates numerous ring-enhancing lesions (green arrows). (d) There is restricted diffusion of the lesions on DWI (red arrow).

**Figure 4:**
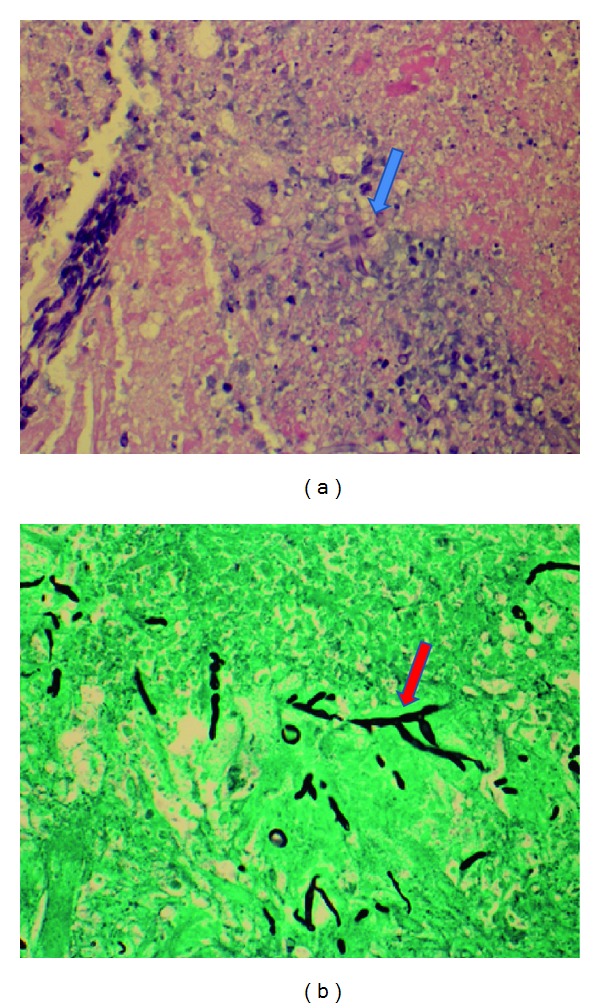
H&E stain (a) reveals hyphal formations (blue arrow) compatible with fungal growth. GMS stain (b) reveals fungal branching (red arrow).

**Figure 5:**
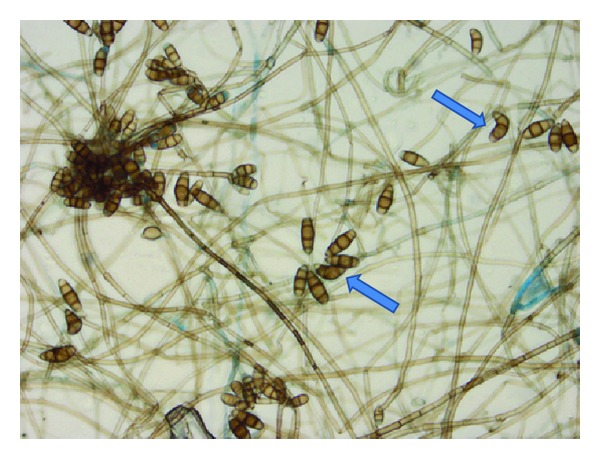
Microscopy reveals conidia, typically with four cells, which eventually appear curved (blue arrows), as the central cell swells as it matures. *Curvularia* spp have brown-pigmented walls, darker central cell than end cells, thin cell wall, and distinct curvature, as fungi develop with age.

The patient presented again fifteen months later with complaints of headache and new onset seizure. The MRI of the brain on this admission revealed numerous intracranial lesions supratentorially. There were five ring-enhancing lesions on the fat suppressed postcontrast images. Initially the differential diagnosis included exacerbation of neurosarcoidosis, intracranial metastasis, or cerebral abscesses. However, each of the intracranial lesions demonstrated restricted diffusion which suggested development of multiple cerebral abscesses (Figure 3). Neurosurgical intervention was successful in debriding the affected areas and resecting the abscesses. The patient was started on intravenous antifungal medications empirically while awaiting culture results. The hematoxylin and eosin (HE) stain in addition to the Grocott methenamine silver (GMS) stain revealed numerous hyphal formations confirming a fungal etiology (Figure 4). The pathology results of the specimen were initially thought to be *Bipolaris* species but subsequently confirmed to be *Curvularia* species as the conidia were found to have a darker central cell than end cells and a distinct curvature due to swelling of the central cell as it develops with age (Figure 5).

**Figure 6:**
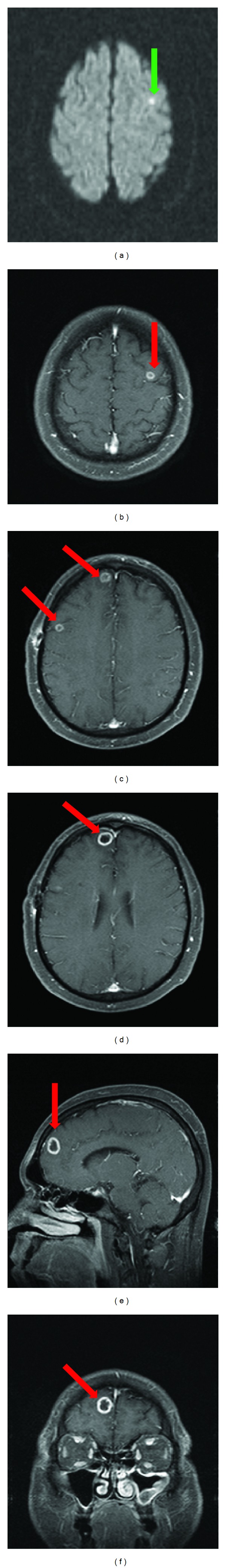
One week followup postsurgical debridement showed new lesions. (a) There is restricted diffusion of the lesions on DWI (green arrow). Axial (b, c, and d), sagittal (e), and coronal (f) T1-weighted fat suppressed images following administration of 15 mL of intravenous gadolinium demonstrate numerous ring-enhancing lesions (red arrows).

**Figure 7:**
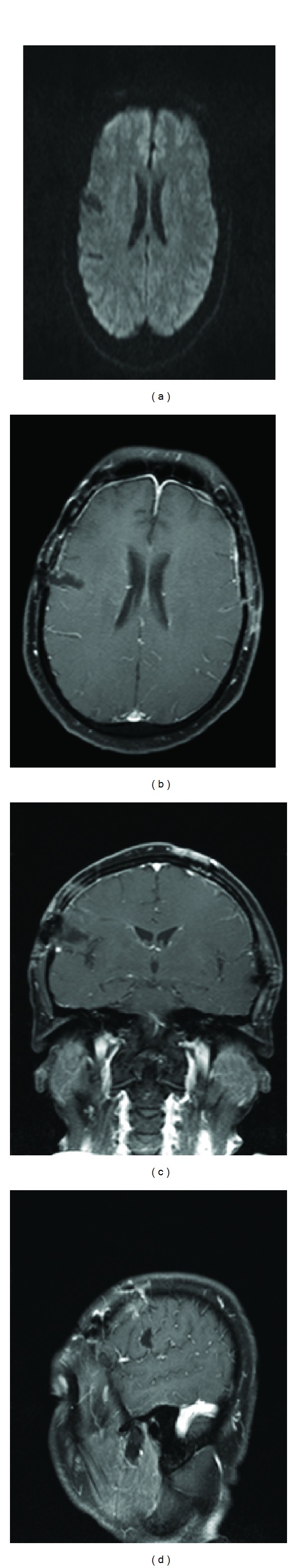
Three-week followup after-second surgical debridement showed no new lesions. (a) There is no restricted diffusion on DWI. Axial (b), coronal (c), and sagittal (d) T1-weighted fat suppressed images following administration of 15 mL of intravenous gadolinium demonstrate resolution of the ring-enhancing lesions. Resection cavity is present in the right frontal cortex.

A one-week followup from the initial surgical debridement showed new abscesses on MRI of the brain (Figure 6), and they were again successfully resected with neurosurgical intervention. The three-week followup from the second surgical intervention showed no new lesions and stable postoperative appearance of the brain. The two-month followup MRI of the brain continued to show no new lesions with stable changes in the brain (Figure 7).

## 3. Discussion

Neurosarcoidosis occurs in approximately 5% of patients with systemic sarcoidosis [1]. The base of the skull is often affected in intracranial neurosarcoidosis with involvement of the basal leptomeninges and dura, as well as the cranial nerves [2]. On imaging, a common manifestation of neurosarcoidosis is parenchymal mass lesions or granulomas which appear as enhancing mass lesions on T1-weighted contrast-enhanced MRI [3]. These lesions can be supratentorial and commonly affect the pituitary gland, infundibulum, and hypothalamus. Since many different structures of the brain can be affected including cranial nerves, neurosarcoidosis presents with a wide spectrum of clinical syndromes [2]. Cranial nerve deficits, however, are the clinical symptoms most frequently encountered with facial nerve palsy being the most common. Additional clinical manifestations of neurosarcoidosis may include encephalopathy, meningitis, or seizures [3]. Patients with sarcoidosis and neurosarcoidosis are treated with chronic steroid therapy [2]. This medical therapy, however, leaves patients immunocompromised and susceptible to various infections.

Cerebral phaeohyphomycoses are mycotic infections from dematiaceous fungi whose cell walls are uniquely darkly pigmented from melanin production. These pigmented molds usually cause disease within the plant ecosystem but occasionally cause localized subcutaneous infections after direct inoculation in humans [4]. These infections are therefore almost exclusively seen in tropical and subtropical regions affecting rural laborers. Disseminated infection and focal visceral infections are exceedingly rare with the typical route of transmission being through inhalation into the respiratory tract, which may subsequently lead to localized infection of the sinuses. Moreover, in immunocompromised patients specifically, this can lead to pneumonia and potentially hematogenous dissemination to the central nervous system [4].

The incidence of cerebral phaeohyphomycosis is increasing and typically carries with it a poor prognosis. Approximately twenty-four fungal species are identified to be neurotropic, of which *Cladophialophora* species and *Rhinocladiella* species are more well known because they are so strongly neurotropic [4–6]. The *Curvularia* species are exceedingly rare causes of cerebral phaeohypomycosis and are limited to case report literature. This syndrome typically manifests as a single brain abscess, while multiple abscesses are seen in immunocompromised patients, as was the case in our patient on chronic steroid therapy for treatment of neurosarcoidosis [7].

Radiographically on MRI studies, cerebral phaeohyphomycoses typically present as ring-enhancing lesions on T1-weighted images, hypointensity of the ring on T2-weighted images, with a centrally low-enhancing area suggesting pus or necrosis [6, 7]. These infections, however, may mimic high-grade glioma or metastasis when there are irregular, variable contrast-enhancing masses [6]. Magnetic resonance spectroscopy (MRS) has been used in addition to magnetic resonance imaging (MRI) to help differentiate the entities [8]. MRI-diffusion-weighted imaging (DWI) and apparent diffusion coefficient (ADC) mapping are established tools in analyzing cerebral fungal abscesses. For instance, cryptococcomas are reported to have a hypointense DWI signal with higher ADC values in these fungal abscesses as compared to the lower values in pyogenic abscesses [8]. Moreover, most glioblastomas do not exhibit restricted diffusion in DWI MRI. Regarding the use of MRS to differentiate intracranial pathology, Hauck et al. suggest that gliomas have an increase of choline with a decrease in N-acetyl-aspartate : choline ratio and increased choline : creatine ratio in high-grade tumors. It is important to note that decreased N-acetyl-aspartate peaks denote loss of neuronal tissue and increased choline peaks are thought to represent cell membrane turn over (e.g., inflammatory reaction). It is helpful to know that in cases where N-acetyl-aspartate : choline ratio is approximately one that the underlying pathology is highly suspicious for glioma and warrants biopsy [8].

The mortality rate of this disease is 100% in the untreated. Even with surgical and chemotherapy intervention, the mortality can be as high as 65% [6]. Thus, early diagnosis with the help of understanding key radiologic features is important. 

## 4. Conclusions

Cerebral phaeohyphomycosis is a rare entity, and infection from *Curvularia* species is exceedingly rare. This particular type of mold commonly infects plants. Fungal sinusitis has previously been attributed to recreational marijuana usage [9]. The mechanism of transmission is most likely secondary to the patient's heavy recreational usage of marijuana. Given the immunocompromised status from chronic steroid therapy for neurosarcoidosis and the clinical setting, the patient most likely developed a fungal pneumonia resulting in the pulmonary abscess seen on the CT of the chest and the most likely nidus for the subsequent hematogenous dissemination resulting in intracranial abscess formation. 

## Abbreviations

CT: Computed tomography
MRI: Magnetic resonance imaging
DWI: Diffusion weighted imaging
ADC: Apparent diffusion coefficient
MRS: Magnetic resonance spectroscopy.
